# Functional Amino Acid Supplementation Drives Early Growth and Gut Maturation in Broilers: A Meta-Analysis

**DOI:** 10.3390/ani16081207

**Published:** 2026-04-15

**Authors:** Emmanuel Nuamah, Utibe Mfon Okon, Jongryun Kim, Guybong Song, Darae Kang, Hakkyo Lee, Kwanseob Shim

**Affiliations:** 1 Department of Animal Biotechnology, Jeonbuk National University, Jeonju 54896, Republic of Korea; 2 Department of Animal Science, Jeonbuk National University, Jeonju 54896, Republic of Korea; 3 Halal Products Research Institute, Universiti Putra Malaysia (UPM), Putra Infoport, Serdang 43400, Selangor, Malaysia; 4 Korea Animal Improvement Association, Jeonju-si 54859, Republic of Korea; 5 Department of Agricultural Convergence Technology, Jeonbuk National University, Jeonju 54896, Republic of Korea

**Keywords:** intestinal stress, immune organs, small intestine, L-arginine, L-glutamine, glycine, broiler chicken, meta-analysis

## Abstract

Early post-hatch feeding strategies supplementing nutrients, particularly functional amino acids, have been proposed to enhance gastrointestinal tract (GIT) maturation and health in broilers in the post-antibiotic era. However, reported effects on performance and gut morphology remain inconsistent. Hence, this meta-analysis synthesized and clarified the efficacy pattern of supplemental FAA (Arg, Gln, Gly) evidence on growth performance, gut morphology, and lymphoid organ development. From a search spanning 2015 to September 2025, data were extracted from 23 eligible studies among 582 reports identified and pooled from five online databases. Standardized mean differences (SMDs) were calculated using Hedges’ *g* estimator with 95% confidence intervals, and heterogeneity was explored using subgroup and meta-regression procedures. Internal validity and reliability of included studies and publication bias were also assessed. The random-effects meta-analyses revealed that the FAA increased BWG (SMD = 1.01; *p* = 0.0006) and reduced feed conversion ratio (SMD = −0.45; *p* < 0.0001). Likewise, they enhanced intestinal architecture in both the jejunum and ileum. This was characterized by increased villus height (*p* < 0.05), reduced crypt depth (*p* < 0.05), and an elevated villus-to-crypt ratio (*p* < 0.0001), with the ileum exhibiting the greatest morphological response. In contrast, supplementation had no significant effect on spleen weight (SMD = 0.24; *p* = 0.2483) or bursa weight (SMD = 0.31; *p* = 0.1575). These effects, however, can be influenced by dosage used, dietary crude protein level, and broiler strain. In addition to enhancing the small intestine morphology early on, longer supplementation increased feed efficiency. Specifically, L-arginine and glycine efficaciously stimulated BWG, while L-glutamine and L-arginine enhanced morphology. Overall, early dietary supplementation with arginine, glutamine, or glycine is an effective post-antibiotic nutritional strategy to alleviate early post-hatch physiological stress and support broiler growth and intestinal development. However, to optimize nutrient utilization and sustain growth performance comparable to that achieved with standard CP diets, these FAAs in practical broiler nutrition should be strategically integrated into low-CP formulations.

## Simple Summary

In the post-antibiotic era of broiler production, nutritional strategies that help maintain gut health are becoming more important. Functional amino acids (FAA), such as L-arginine, L-glutamine, and glycine, have been studied because they may help reduce intestinal stress when provided at levels above normal dietary levels. However, their effects on gut structure and growth performance have been inconsistent across studies. Therefore, this study used a meta-analysis to evaluate the overall effects of these amino acids and to determine the production conditions in which they are most beneficial. The findings aim to support more sustainable and efficient feeding strategies for modern broiler production.

## 1. Introduction

Commercial poultry production has, over the decades, evolved into an integral component of the global food system, serving as a major contributor to the supply of high-quality animal protein [1,2]. To meet the future demand [3], driven by the growing global population, the industry’s efficiency to convert feed into animal protein has been linked to the quality of day-old chicks at placement [4]. In particular, it is highlighted as crucial to optimal growth rates and survival up to slaughter age in broiler production [5,6,7]. Though broiler lines have been genetically selected for enhanced body weight gain, feed efficiency, and breast muscle yield [8,9], the conditions in the egg and in commercial hatcheries affect the development of the embryo, which reduces hatchability and chick quality [4,10,11]. This, in turn, shapes how the bird performs in the long term.

Regardless of the rapid growth potential, the modern strains of broilers remain highly susceptible to early post-hatch physiological stress that impedes the functional maturation of the gastrointestinal tract (GIT) [12,13,14]. This stress possibly alters the GIT’s primary role to convert feed nutrients into body mass [15], which is key to gaining optimal performance. To support, restore, or maintain optimal GIT homeostasis [16,17], numerous nutritional and nutrient interventions have been proposed and evaluated in the post-antibiotic era [18,19,20]. Among these, the supplementation of functional amino acids (FAA), including methionine, arginine, proline, glutamine, leucine, glycine, tryptophan, and cysteine, has received considerable attention in poultry nutrition [21].

For example, dietary supplementation of conditionally essential amino acids, such as glutamine and glycine, as well as arginine, an indispensable amino acid in poultry, beyond levels provided by conventional diets, has been demonstrated to optimize growth performance in young animals [22,23,24] and prevent disease [25]. These FAAs (Arg, Gln, Gly) play central roles in multiple signaling pathways related to intestinal stress, thereby regulating gene expression, hormone regulation, energy metabolism, and the precursors of functional molecules and proteins [26,27]. Overall, they regulate muscle growth and regeneration, immune competence, development, endocrine activity, and antioxidant defense [28,29,30,31]. Growing evidence suggests that young animals, including broilers, cannot synthesize sufficient amounts of these amino acids to support maximum embryonic survival, neonatal growth, and vascular and intestinal health [32,33,34]. As a result, their supplementation, either in standard protein or low crude protein (Low-CP) formulations, is now a common practice in broiler nutrition [35]. However, some studies of their supplementation have reported varied outcomes, ranging from beneficial to negligible or even adverse effects on growth and health [36,37,38,39,40,41,42], leading to ongoing debate in the field. Addressing these discrepancies is critical, as they carry significant implications for feed formulation strategies, raw material utilization, and the sustainability of poultry production systems.

Dietary characteristics influencing the response to these functional amino acids remain heterogeneous [43], particularly the lack of a standardized low-crude-protein diet, which has mainly adopted a gradual reduction or replacement of the protein source, such as soybean meal. In addition, variant baseline characteristics, such as dietary characteristics, feeding management, environment, dosage, strain, sex, and growth phase [43,44], continue to limit its statistical power. To address the issue of statistical power, meta-analysis, a data mining procedure (of a new empirical model), has been proposed [45] and applied in broiler nutrition to evaluate the effects of FAA [43,46,47]. However, existing analyses have predominantly focused on growth performance or feed efficiency, often under specific experimental conditions such as pathogen challenge, heat stress, or low crude protein diets, either evaluated independently or in combination with other amino acids. To date, no meta-analysis has explored the FAA’s effects on gut morphology and immune organs or characterized the contexts in which these FAAs exert their effects.

Therefore, the objectives of the present meta-analysis were twofold: (i) to synthesize and clarify the efficacy pattern of post-hatch dietary supplementation with L-arginine, L-glutamine, and glycine on growth performance, gut morphology, and the relative development of lymphoid organs; and (ii) to identify and investigate the sources of between-study variation across effect sizes through subgroup and meta-regression analyses.

## 2. Materials and Methods

This meta-analysis was conducted in accordance with the Preferred Reporting Items for Systematic Reviews and Meta-Analyses (PRISMA) guidelines. Only animal data derived from previously published experimental studies were included. As no new animal experiments were performed, approval from institutional animal care and use committees was not required.

### 2.1. Database Search and Screening Procedures

Search keywords, including broiler chicken, arginine, glutamine, glycine, growth performance, and gut morphology, were formulated based on the PICO framework [48], comprising the following components: Population (broiler chickens), Interventions (basal diet supplemented independently with arginine, glutamine, or glycine), Comparator (basal diet without supplementation), and Outcomes (growth performance, gut morphology, and lymphoid organ development). To identify studies addressing the research question, “What is the modulatory role of functional amino acids (arginine, glutamine, and glycine) on early post-hatch intestinal stress in relation to broiler growth, gut, and immune organ responses?”—a comprehensive literature search was conducted. The search strategy covered publications from 2015 to September 2025 and included five electronic databases: Web of Science, Scopus, ScienceDirect, PubMed, and Google Scholar, all accessed on 17 September 2025.

**Figure 1:**
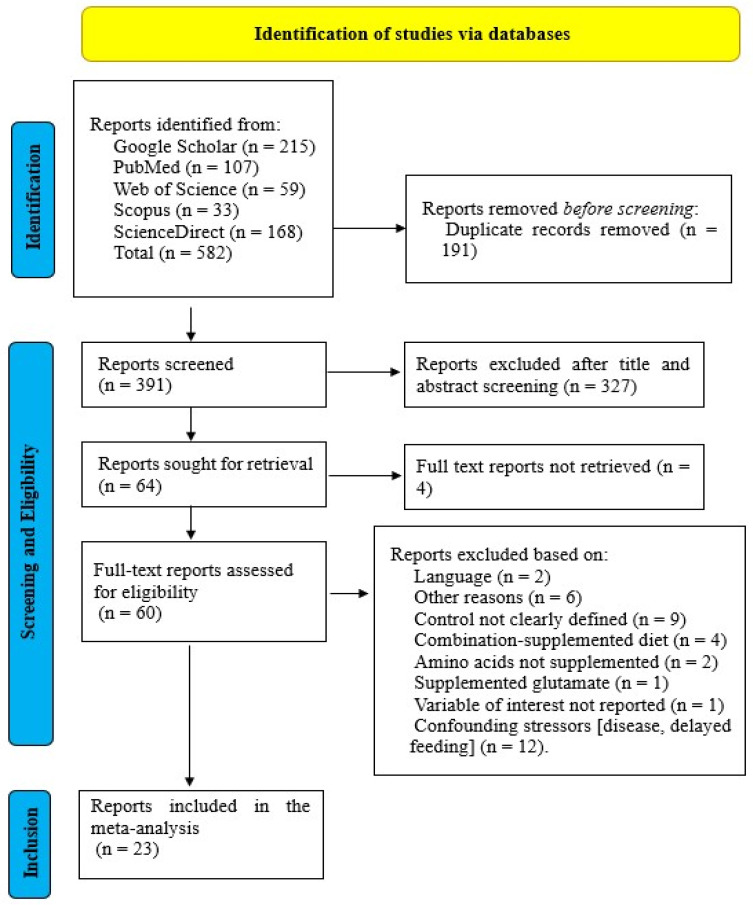
PRISMA flow diagram summarizing study identification, screening, and inclusion.

A total of 582 potentially relevant records were initially identified and pooled. After removing 191 duplicate entries using Zotero (Version 8.0-beta.8), 391 unique records remained. To ensure metadata integrity and minimize selection bias, two independent reviewers conducted a rigorous two-step screening process, as outlined by Nuamah et al. [49] and illustrated in Figure 1.

### 2.2. Criteria for Inclusion and Exclusion

In the second phase of the two-step screening process, studies that passed the initial title and abstract screening were assessed for eligibility based on the meta-analysis’s predefined inclusion and exclusion criteria. Inclusion criteria were as follows: (1) use of one-day-old broiler chicks sourced from hatcheries; (2) peer-reviewed articles published in English; (3) a control basal diet; (4) intervention group: basal diet supplemented independently with arginine, glutamine, or glycine (5) reporting of at least one of the variables of interest; (6) clear randomization of broiler allocation; (7) quantification of the supplemented amino acid dose; and (8) provision of mean values, variability measures (SD or SEM), and sample sizes for both control and intervention groups.

Studies were excluded if they: (1) involved confounding stressors such as disease or delayed feeding; (2) lacked a clearly defined control group; (3) fed basal diet supplemented with a combination of the evaluated amino acid as intervention; (4) used glutamate instead of glutamine; (5) failed to report variables of interest; (6) were published in languages other than English; (7) had inaccessible full texts; or (8) other unspecified reasons. In total, 23 full-text articles satisfied the eligibility criteria and were subsequently included in the meta-analysis, as illustrated in Figure 1.

### 2.3. Data Extraction and Processing

A database was constructed using structured spreadsheets in Google Sheets (Google LLC, Mountain View, CA, USA). From each eligible study, two categories of information were extracted: (i) study characteristics and design features (author, year, country, broiler strain, sex, growth phase, number of birds per treatment, basal diet type, calculated CP percentage of the basal diet, basal diet’s CP formulation type, amino acid type and dosage used, and duration of supplementation), as summarized in Table 1; and (ii) reported outcomes, including mean values for growth performance parameters (ADFI, BWG, and FCR), intestinal morphometrics of the jejunum and ileum (villus height [VH], crypt depth [CD], and VH-to-CD ratio), and relative lymphoid organ weights (spleen and bursa of Fabricius), along with corresponding sample sizes and measures of variability (SD). Studies that included more than one dose level of the intervention group were considered separate experimental comparisons relative to the control group. The compiled dataset was subsequently organized in Microsoft Excel (Version 2508, Microsoft Corp., Redmond, WA, USA) and used for meta-analysis. However, due to insufficient records, a reliable meta-analysis of duodenal morphology and the thymus organ could not be conducted, so these variables were excluded from the results.

### 2.4. Risk of Bias Assessment

**Figure 2:**
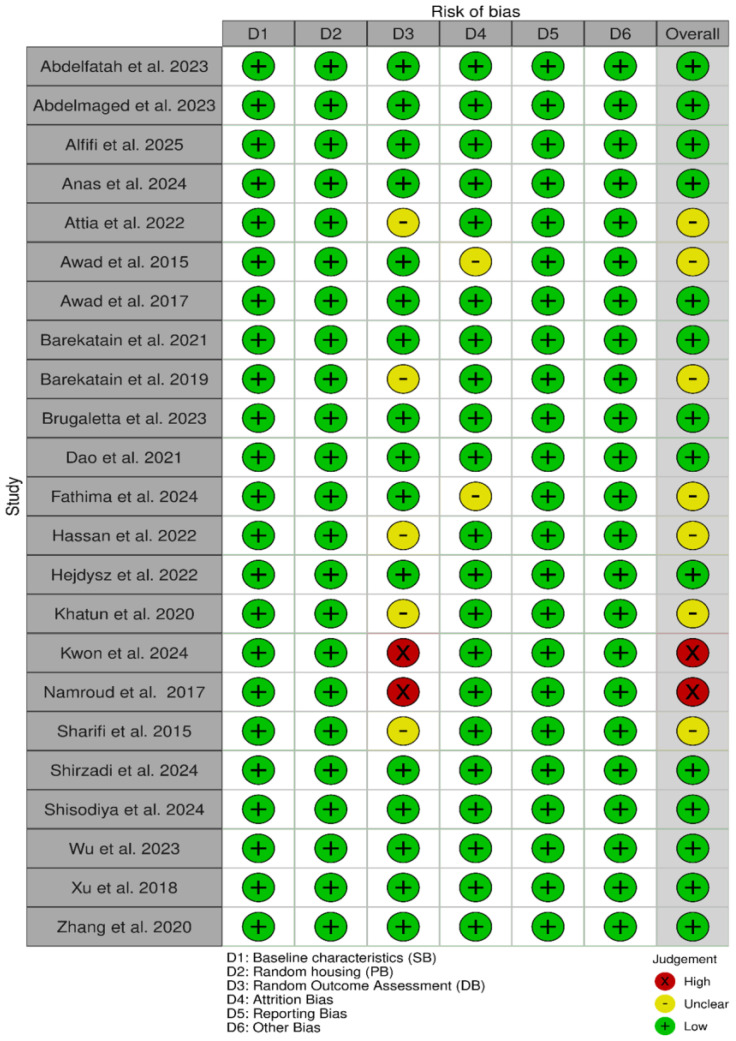
“Traffic light” plots of the domain-level judgements for each individual report.

Following the Cochrane Collaboration’s checklist for animal studies, the internal validity of each report included in the meta-analysis was assessed using the Systematic Review Center for Laboratory Animal Experimentation (SYRCLE) risk of bias tool [70]. Each study was rated on a three-point scale (low risk, high risk, or unclear) across the following domains: baseline characteristics, random housing, random outcome assessment, incomplete outcome data, selective outcome reporting, and other potential sources of bias. The domains of sequence generation, allocation concealment, and blinding (performance and detection) were excluded, as these domains are not yet standard reporting practice in animal experimentation studies [49,71,72]. The domain-level judgements for each report rating and overall risk of bias were subsequently summarized and visualized using “traffic light” plots (Figure 2) and weighted bar plots (Supplementary Figure S1) generated with the Robvis tool [73].

### 2.5. Statistical Analysis

All statistical analyses were conducted in R version 4.5.2 (R Core Team) using the *meta* and *metafor* packages.

#### 2.5.1. Model Selection, Effect Measure, and Meta-Analysis

A random-effects model meta-analysis was conducted to estimate the overall effect sizes across studies. Standardized mean differences (SMDs), weighted by the inverse-variance method, with corresponding 95% CIs, were computed for the registered continuous data of the control and amino acid-supplemented groups, using Hedges’ *g* estimator to account for differences in measurement scales across studies. According to Cohen [74], SMDs of 0.2–0.5, 0.5–0.8, and above 0.8 indicate small, medium, and large effect sizes, respectively. Forest plots were used to show individual study SMDs, overall effect size, and prediction interval.

#### 2.5.2. Publication Bias and Trim-Fill Procedure

Publication bias, or the small-study effect, was visualized using contour-enhanced funnel plots. This was confirmed through numerical assessment using Begg’s rank correlation method, with statistical significance defined as *p* ≤ 0.05. To ensure the robustness of the meta-analysis findings of outcome variables with small-study effects, correction analyses were performed using Duval and Tweedie’s trim-and-fill procedure [75]. This approach estimated the number of potentially missing studies and recalculated the overall effect size to account for publication bias.

#### 2.5.3. Heterogeneity and Variance Estimation

Between-study variance (τ^2^) was estimated using the restricted maximum likelihood (REML) method to evaluate the consistency of effects across studies [76]. Heterogeneity was assessed using the chi-squared (Q) test, with significance set at *p* ≤ 0.10 [77]. The proportion of total variation attributable to heterogeneity was quantified using the I^2^ statistic, categorized as low (<25%), moderate (25–50%), high (50–75%), and very high (75–100%). Sensitivity analyses were conducted to determine the influence of potential outliers on pooled effect sizes and heterogeneity estimates. These were visualized using Cook’s distance, difference-in-fit plots derived from leave-one-out analyses, and Baujat plots. Outcomes that continued to exhibit moderate heterogeneity (Q test *p* < 0.01; I^2^ ≥ 50%) were further investigated using subgroup analysis and meta-regression to identify potential sources and patterns of variation.

## 3. Results

This meta-analysis synthesizes and critically evaluates the efficacy of post-hatch independent supplementation of FAA (L-arginine, L-glutamine, and glycine) in standard and low-CP diets. Broiler responses to these amino acids, investigated as nutritional interventions at varying inclusion levels, were assessed using random-effects models. Outcomes included growth performance, jejunal and ileal morphometric characteristics, and lymphoid organ response.

### 3.1. Risk of Bias Assessment

Domain-specific risk-of-bias assessments for the included studies, along with the weighted distribution across domains in the meta-analysis, are presented in Figure 2 and Supplementary Figure S1, respectively. Studies [64] and [65] were judged to have a high risk of bias (Figure 2, traffic light plots). However, the weighted distribution (Supplementary Figure S1) indicates that all studies were consistently rated as low risk concerning baseline characteristics, random housing, reporting bias, and other potential sources of bias. Although high risk related to random-outcome assessment may influence the direction or magnitude of effect estimates, sensitivity analyses using the Baujat plot and the leave-one-out procedure did not identify these studies as influential outliers. Overall, the body of evidence demonstrated strong methodological rigor, with 61% of studies assessed as low risk of bias and 30% as unclear risk. The predominance of low-risk assessments supports the robustness, validity, and reliability of the meta-analytic findings.

### 3.2. Meta-Analysis of Global Studies

#### 3.2.1. Growth Performance

**Figure 3:**
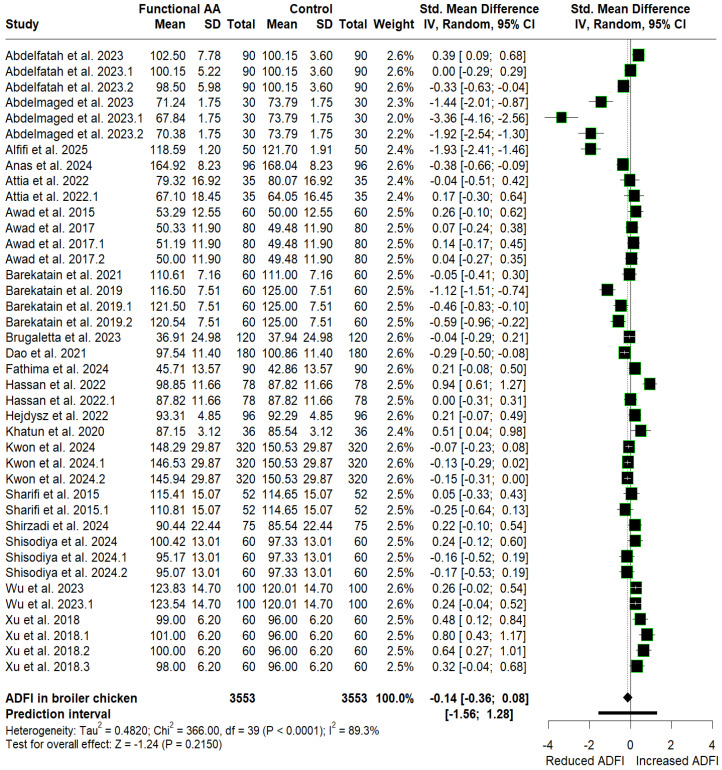
Forest plot depicting the SMD and corresponding 95% CI for the effects of post-hatch L-arginine, L-glutamine, and glycine supplementation on ADFI in broilers.

**Figure 4:**
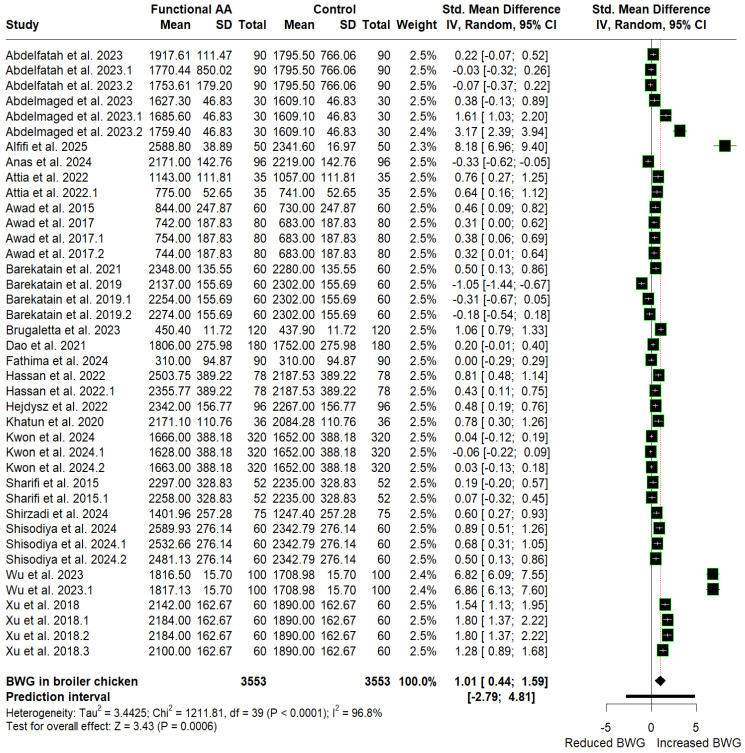
Forest plot showing the SMD and 95% CI for post-hatch L-arginine, L-glutamine, and glycine supplementation effects on BWG in broilers.

**Figure 5:**
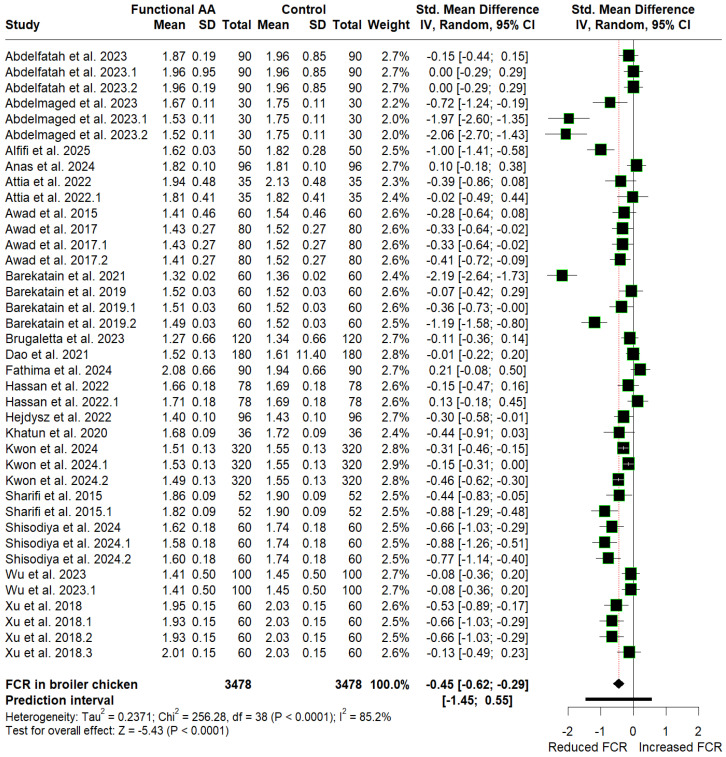
Forest plot showing the SMD and 95% CI for the effects of post-hatch L-arginine, L-glutamine, and glycine supplementation on FCR in broilers.

The mean values of ADFI, BWG, and FCR for the control and amino acid supplementation groups, along with their SMD estimates, are presented as forest plots in Figure 3, Figure 4 and Figure 5, respectively. Supplementation with FAA (arginine, glutamine, and glycine) favored reductions in ADFI (SMD = −0.14, *p* = 0.215, 95% CI: −0.36 to 0.08) and FCR (SMD = −0.45, *p* < 0.0001, 95% CI: −0.62 to −0.29), corresponding to a small effect, respectively, compared with the control group. In contrast, supplementation significantly increased cumulative BWG (SMD = 1.01, *p* = 0.0006, 95% CI: 0.44 to 1.59), equivalent to a large effect. Although supplementation significantly improved BWG and FCR, heterogeneity remains high across studies (I^2^ ≥ 50% with *p* < 0.10), suggesting that other additional underlying factors may influence the observed responses. Furthermore, because the prediction intervals span zero (BWG: −2.79 to 4.81; FCR: −1.45 to 0.55), future applications could result in either performance enhancements or reductions.

Evidence of publication bias was found exclusively in the BWG and FCR datasets, as indicated by funnel plot asymmetry (Supplementary Figure S10A,B) and confirmed by Begg’s tests (Table 2). However, the direction of the pooled effect sizes for BWG (SMD = 0.14, *p* = 0.687; 95% CI: −0.54 to 0.82) and FCR (SMD = −0.23, *p* = 0.030; 95% CI: −0.44 to −0.02) remained unchanged after adjusting for potentially missing studies using the trim-and-fill method (Table 2). Therefore, the observed asymmetry does not materially alter the interpretation of the findings, confirming that the conclusions of our initial meta-analysis remain robust.

#### 3.2.2. Jejunal and Ileal Morphometric Characteristics

The overall SMD estimates for FAA effects relative to the control group on jejunal and ileal morphometric characteristics are summarized in Table 3 (forest plot; Supplementary Figures S2–S7). Supplementation with L-arginine, L-glutamine, and glycine significantly increased jejunal villus height (SMD = 1.06, *p* = 0.003, 95% CI: 0.36 to 1.77) and the villus height-to-crypt depth (VH-to-CD) ratio (SMD = 1.05, *p* < 0.0001, 95% CI: 0.64 to 1.45) in broilers fed either standard or low-CP formulations. Conversely, crypt depth was significantly reduced (SMD = −0.97, *p* = 0.0001, 95% CI: −1.46 to −0.47). These morphological adaptations may have increased absorptive surface area, reduced epithelial turnover, and improved nutrient utilization.

Between-study heterogeneity was generally low (I^2^ < 25%) for ileal morphology indicators but remained moderate to high (I^2^ = 25–75%) for the jejunal response. Although funnel plot asymmetry (Supplementary Figures S10C–E) and Begg’s test (Table 3) indicated publication bias across all jejunal outcomes, trim-and-fill adjustments for potentially missing studies did not alter the direction of the initial estimates. Post-adjustment pooled effect sizes remained stable and significant for both CD (SMD = −0.77, *p* = 0.012; 95% CI: −1.38 to −0.17) and the VH-to-CD ratio (SMD = 0.84, *p* = 0.001; 95% CI: 0.33 to 1.34).

Likewise, supplementation increased both ileal VH (SMD = 1.10, *p* = 0.002; 95% CI: 0.51 to 1.69) and the VH-to-CD ratio (SMD = 1.07, *p* < 0.001; 95% CI: 0.55 to 1.58) and reduced crypt depth (SMD = −0.54, *p* = 0.022; 95% CI: −1.01 to −0.08). The effect, however, is more robust and consistently pronounced in ileal morphology, whereas jejunal CD shows a stronger isolated response, suggesting a potentially greater ileal sensitivity. Collectively, these findings suggest that the growth-promoting effects of these FAAs are not confined to a single intestinal segment.

#### 3.2.3. Relative Lymphoid Organ Weight

The effects of arginine, glutamine, and glycine supplementation on the relative weights of the spleen and bursa of Fabricius are detailed in Table 3. Relative to controls, FAA supplementation did not significantly alter the weights of the spleen (SMD = 0.24, *p* = 0.248; 95% CI: −0.16 to 0.64) or the bursa of Fabricius (SMD = 0.31, *p* = 0.158; 95% CI: −0.12 to 0.73). Notably, these outcomes exhibited no significant between-study heterogeneity or publication bias (Table 2). However, given the width of the prediction intervals, the overall direction of the effect remains uncertain, suggesting that future studies may observe either an increase or a decrease in relative lymphoid organ weights.

### 3.3. Variation in Growth Performance and Jejunal Villus Height Across Subgroups

Recognizing that the studies included in the meta-analysis represent multiple subgroups rather than a single homogeneous population, each with potentially distinct overall effects, subgroup analyses were performed to test the hypothesis that supplementation with arginine, glycine, and glutamine, amino acids known for their roles in immune function, gut morphology, and epithelial proliferation, respectively, exerts differential effects. An overview of subgroup-specific variability in ADFI, BWG, FCR, and jejunal VH is presented in Table 4, Table 5, Table 6 and Table 7.

#### 3.3.1. Amino Acid Type

The type of amino acid supplemented significantly explained the observed heterogeneity for broiler BWG (P_subgroup_ = 0.0185, R^2^ = 10.6; Table 5), FCR (P_subgroup_ = 0.0002, R^2^ = 31.2; Table 6), and jejunal villus height (P_subgroup_ = 0.0130, R^2^ = 27.1; Table 7) but not ADFI (Psubgroup = 0.5361, R^2^ = 0.0; Table 4). Supplemental arginine and glycine improved BWG and FCR, making them the most notable drivers of growth performance. Glutamine and, to a lesser extent, arginine enhanced jejunal VH. This suggests that while arginine and glycine excel in promoting growth, glutamine is more specifically associated with maintaining intestinal morphology.

#### 3.3.2. Type of Crude Protein Formulation

The CP formulation type partly explained variation in the efficacy of arginine, glycine, and glutamine supplementation on BWG (P_subgroup_ = 0.0037, R^2^ = 15.9; Table 5) and jejunal VH (P_subgroup_ = 0.0423, R^2^ = 12.8; Table 7). Although supplementation consistently improved BWG across both standard- and low-CP formulations, the magnitude of this benefit, along with the pronounced improvement in jejunal VH, was maximized under standard CP conditions. Therefore, while the benefits of these FAAs are most evident in standard diets, their strategic supplementation in low-CP diets still helps mitigate nutritional stress. 

#### 3.3.3. Growth Phase

The growth phase at the time of supplementation accounts for some of the variation in ADFI (P_subgroup_ = 0.0162, R^2^ = 4.6; Table 4), FCR (P_subgroup_ = 0.0113, R^2^ = 2.8; Table 6), and jejunal VH (P_subgroup_ = 0.0423, R^2^ = 12.8; Table 7). While supplementation during the early growth phase provided the greatest benefits for intestinal morphology, improvements in feed efficiency were far more pronounced in longer-term studies. Notably, the reduction in FCR was maximized during the grower phase (≥21 days), indicating that the utilization benefits of these FAAs accumulate and intensify over time.

#### 3.3.4. Broiler Strain

Broiler strain significantly influences the effects of FAA supplementation, partially explaining the variation in ADFI (P_subgroup_ < 0.0001, R^2^ = 17.1; Table 4), BWG (P_subgroup_ < 0.0001, R^2^ = 14.2; Table 5), FCR (P_subgroup_ < 0.0001, R^2^ = 12.2; Table 6), and jejunal VH (P_subgroup_ = 0.0175, R^2^ = 25.2; Table 7). These results highlight that a bird’s genetic background strongly influences its response to targeted nutritional interventions. Notably, while the Arbor Acres, Cobb-430Y, and Ross 308 strains exhibited the most pronounced improvements in BWG and FCR, the Ross 308 strain showed the most sensitive response for ADFI and jejunal VH.

### 3.4. Meta-Regression: Growth Performance and Jejunal Villus Height

The meta-regression analysis examining amino acid dosage used and duration supplemented and the calculated chemical composition CP level of the basal diet is presented in Table 8. The results indicate that the effectiveness of amino acid supplementation on growth performance and jejunal villus height is context-dependent, varying according to these moderating factors.

#### 3.4.1. Amino Acid Dose

The dose of Arg, Gln, or Gly supplemented did not moderate the amino acid’s impact on BWG or FCR in broilers. Nonetheless, it predicted an increase in jejunal VH (P_QM_ = 0.0510) and yielded a borderline moderation effect on ADFI (P_QM_ = 0.0649). A higher dose was associated with a positive trend in both metrics, accounting for 6.00% and 37.50% of the heterogeneity in ADFI and VH, respectively. This suggests that increasing the amino acid dose may show a strong trend towards enhancing gut morphology, as measured by jejunal VH.

#### 3.4.2. Supplementation Duration

Unlike ADFI, BWG, and jejunal VH, the duration of Arg, Gln, or Gly supplementation significantly moderates its impact on FCR (P_QM_ = 0.0396, R^2^ = 9.00%). Thus, a more extended supplementation period enhances the feed nutrient utilization efficiency (promoting a decrease in the birds’ FCR).

#### 3.4.3. Calculated Crude Protein Level

The formulation’s CP level significantly moderates the FAA’s impact on BWG (P_QM_ = 0.0019, R^2^ = 18.70). Thus, confirming our subgroup analysis, the effects of the evaluated FAA were substantially larger in studies using a standard CP formulation than in those using a low-CP formulation. The estimate (0.4091, Table 8) indicates that as the CP% in the diet increased, the effect of the evaluated FAA on BWG also increased. In addition, explaining 20% of the observed heterogeneity, the dietary CP% significantly (P_QM_ = 0.0553) moderates the FAA’s impact on increasing jejunal VH. This affirms our results that dietary protein level influences the effect of the Arg, Gln, or Gly on increasing jejunal VH.

## 4. Discussion

Efforts to improve the efficiency of poultry production and mitigate its environmental impact have increasingly focused on nutritional strategies, particularly the development of low-CP diets and precision feeding approaches through supplementation with cost-effective crystalline amino acids [78]. Beyond their fundamental role in protein synthesis and growth optimization, amino acids have been investigated for additional functional properties, including intestinal protection, immune modulation, microbial regulation, and overall enhancement of poultry performance [29]. Despite nearly a century of research elucidating their effects on broilers, contradictions remain. To address these discrepancies, our meta-analysis, comprising a wide range of studies with diverse dietary formulations and intrinsic factors such as broiler strain, sex, and age, aims to provide novel insights into the potential application of Arg, Gln, or Gly in broiler nutrition.

### 4.1. Growth Performance

Advances in poultry genetics and management practices have reshaped the nutritional requirements of modern poultry, creating opportunities to increase the dietary concentration and commercialization of feed-grade functional amino acids beyond meeting the requirements for methionine, lysine, and threonine. Supplementation with these functional amino acids supports gastrointestinal integrity, immune function, behavior, and sustainability [79]. Under conditions of heat or intestinal stress in broilers, such supplementation further improves muscle growth, nutrient efficiency, oxidative stress resistance, and intestinal health [80,81,82].

Our meta-analysis corroborated this assertion and previous reports of enhanced growth performance in broilers [83,84,85,86], demonstrating that supplementation with FAA (arginine, glutamine, and glycine) improves BWG and FCR. Among these, arginine and glycine exerted more precise effects than glutamine (Table 5 and Table 6), which could be attributed to their direct and synergistic involvement in energy metabolism and muscle development. These benefits may, in part, be ascribed to their collaborative role as precursors of creatine [87,88].

Creatine, synthesized in vertebrates [88], serves as a critical energy reservoir for muscle tissue [89] and could have accounted for a substantial proportion of arginine and glycine utilization. A possible explanation for the significant positive response to increasing arginine and glycine on FCR, which translates to better BWG, is that both act as readily available precursors for methylation of guanidino acetic acid (GAA), which is an essential metabolic intermediary product to promote the synthesis of creatine in muscle tissues [90]. This assertion is supported by the position of DeGroot et al. [91] and Lemme et al. [92] that elevated creatine concentrations in pectoral muscles enhance nutrient efficiency for growth. Intrinsically, creatine supports adenosine triphosphate (ATP) resynthesis during cellular energy metabolism, facilitating protein accretion and muscle hypertrophy [93]. It is also worth noting that arginine and glycine support for BWG in reduced-protein diets (Table 5) could be facilitated by their sparing effects of methionine. This consequently redirects the methionine to be used for protein deposition without it being drained for unnecessary synthesis of GAA and creatine [94]. Thus, arginine and glycine supplementation are demonstrated as effective strategies to improve growth performance, carcass yield, lean meat deposition, and skeletal development [84,95,96].

In addition to creatine synthesis, improvements in BWG and FCR may have involved other metabolic pathways, including nitric oxide (NO) synthesis, uric acid metabolism, glutathione production, and protein turnover [97,98]. Arginine, via the urea cycle, and glycine, via uric acid synthesis, represent primary routes of nitrogen excretion in avian species. Arginine-derived NO could further enhance muscle perfusion, improving nutrient and oxygen delivery to support optimal growth [99] through NO as a cytotoxic mediator of immune cells [100].

Though not directly involved in the fundamental processes of muscle protein accretion, such as arginine and glycine, our findings clearly show the beneficial effects of glutamine on broiler BWG. Consistent with our observation, numerous studies have shown that glutamine acts as a principal energy source for rapidly dividing cells, particularly enterocytes lining the GIT and various immune cells [101,102,103,104]. In chickens, due to the limited activities of arginase [105] and proline oxidase [106] in glutamine synthesis from arginine and proline, dietary glutamine is particularly important for bird growth, particularly under stress conditions [107]. In support of this position, Lee, Chang [108], studying the development of weaned piglets, demonstrated that glutamine plays an essential role in muscle structure, body tissues, and BWG. Similarly, glutamine or feed-grade glutamine supplementation in broiler chickens stimulated muscle protein synthesis and whole-body growth [109].

Although beneficial effects of arginine, glycine, and glutamine were observed, the response (BWG and FCR) of broilers to these FAAs was moderated by broiler strain, age, and dietary factors, including crude protein concentration in the diet, and duration of supplementation. These findings establish and confirm that the contradiction of results of supplemental arginine, glycine, and glutamine studies, below, at or above the recommended level, and their variable influence on broiler chicken growth performance could be contextualized to factors, including age, sex, and the strain of birds [44].

### 4.2. Intestinal Morphology and Relative Lymphoid Organ Weight

Variation in broiler response to supplemental Arg, Gln, or Gly suggests that the dietary requirements for maximizing growth and tissue buildup may differ from those needed to support health and immune function [42]. In relation to immune competence, the immaturity of the GIT renders broilers particularly vulnerable to digestive disturbances during early development [110]. As a pointer to the small intestine’s regenerative and absorptive capacity, the evidence synthesis revealed that L-glutamine was particularly efficient for promoting the jejunum and ileum’s morphology. However, the benefit was pronounced in the early phase of growth with standard CP diets and was influenced by the FAA dose.

Our observation confirms the findings of previous original studies, which reported that supplemental glutamine and arginine can increase villus height, decrease crypt depths, and improve the ratio of villus height to crypt depth in birds at normal health status and under enteric challenge [40,111,112,113,114,115,116,117]. Improved VH and VH-to-CD ratios are well established as indicators of enhanced growth performance, primarily due to favorable modifications in the small intestinal morphology [118,119,120]. These effects are especially pronounced (Table 3) in the ileum, likely due to the segment’s higher adaptive potential and its role in acting as a secondary checkpoint for nutrient recovery and barrier defense, where the supplemented FAA might have provided a more significant “trophic boost” [40,113,121,122].

These effects may be partly connected to glutamine’s and arginine’s involvement in polyamine synthesis (1-arginase pathway) and energy homeostasis, respectively. Polyamines (putrescine, spermine, and spermidine) possibly increased lymphocyte mitogenesis and arginine-dependent macrophage-mediated tumor cell cytotoxicity [123]. These may have played a vital role in the development of the small intestine mucosa by promoting cell proliferation and tissue growth [124]. On the other hand, glutamine may have been used as a substrate for the production of nucleic acids, nucleotides, adenosine triphosphate, nicotinamide adenine dinucleotide phosphate, and CO_2_ [125], thereby favoring increased VH and a higher VH-to-CD ratio. Glutamine enhancement of the small intestinal morphology may also be associated with signaling activity, increasing mitochondrial functional integrity by activating heat shock protein 72 (HSP72) expressed by enterocytes [126]. The nitric oxide pathway, a product of arginine, that stimulates macrophage production, could also partially enhance gut development and nutrient absorption in the small intestine [114]. Our findings show that the evaluated FAA supplementation did not significantly affect lymphatic organ growth in broilers, indicating that it may not hinder immune organ development.

Despite the robustness of our findings, the meta-analysis was constrained by limited statistical power in some moderator subgroups, particularly those with high between-study variability (I^2^ ≥ 50%), and should be interpreted with caution. A notable example is the early growth phase (≤21 days), where the scarcity of standardized trials reduces confidence in the isolated long-term performance effects of the evaluated FAA. This highlights the need for more rigorously designed and harmonized early-phase studies to strengthen the precision and interpretability of future pooled estimates. A further key limitation relates to fewer trials of L-glutamine and the strains of broiler represented in the included studies. The subgroup of strains, such as Cobb-430Y, Ross, and Ross × Ross, limits the generalizability of the findings to the broader genetic and functional diversity of modern broiler populations.

## 5. Conclusions

The present synthesis of evidence confirms that dietary supplementation with L-arginine, L-glutamine, and glycine is an essential post-antibiotic-era nutritional intervention to mitigate the post-hatch physiological stress in broiler chickens. These amino acids improve BWG and FCR, with pronounced effects observed during the early growth phase and in standard crude protein formulations. In addition, without compromising lymphoid organ immunity, the supplementation of the evaluated FAA consistently preserved the morphologies of the jejunal and ileal segments of the small intestine. The nutrient utilization and small intestinal morphology-modulatory effects of the evaluated FAA, however, can be influenced by its dosage and supplementation duration and, to some extent, by the growth phase, broiler strain, and dietary crude protein level. Overall, the findings suggest that early dietary supplementation of these amino acids supports broiler growth and improves intestinal development. However, to optimize nutrient utilization and sustain growth performance comparable to that achieved with standard CP diets, these FAAs in practical broiler nutrition should be strategically integrated into low-CP formulations.

## Figures and Tables

**Table 1:** Intrinsic attributes of the studies incorporated into the meta-analysis.

Study	Country	Weighted CP, DM ^a^	CP Formulation	Diet Type	Strain	Sex	Growth Phase, d	^b^ Amino Acid Type	Dose, g/kg Feed	Supplementation Duration
[29]	Egypt	20.74	Standard	Corn-soybean	Arbor Acres	Male	≥21	Glycine	2.5, 1.7, 0.8	35
[50]	Egypt	21.37	Standard	Corn-soybean	Arbor Acres	Unsexed	≥21	L-Arginine	1, 3, 5	38
[51]	Egypt	20.78	Standard	Corn-soybean	Ross	Male	≥21	L-Arginine	1.5	35
[52]	USA	16	Low-CP	Corn-soybean	Cobb 500	Female	≥21	L-Arginine	2	24
[53]	Saudi Arabia	18.29	Low-CP	Corn-soybean	Arbor Acres	Male	≤21 & ≥21	L-Arginine	2.3, 3.9	21, 28
[54]	Malaysia	16.21	Low-CP	Corn-palm oil-soybean	Cobb 500	Male	≤21	Glycine	8.1	21
[55]	Malaysia	16.21	Low-CP	Corn-palm oil-soybean	Cobb 500	Male	≤ 21	Glycine	8.3, 10.03, 12.3	21
[56]	Australia	14.85	Low-CP	Corn-wheat-soybean	Ross 308	Male	≥21	L-Arginine	5	28
[57]	Australia	18.54	Low-CP	Wheat-sorghum-soybean	Ross 308	Male	≥21	L-Gln, Gly, L-Arg	10, 10, 5	28
[58]	Italy	22.8	Standard	Corn-wheat-soybean	Ross 308	Male	≤21	L-Arginine	1.5	14
[59]	Australia	18.96	Low-CP	Wheat-sorghum-soybean	Ross 308	Male	≥21	L-Arginine	2.9	28
[60]	USA	22.8	Standard	Corn-DDGS-soybean	Cobb-500	Male	≤21	L-Arginine	16.56	14
[61]	Bangladesh	17.83	Low-CP	Corn-soybean	Ross × Ross	-	≥21	L-Arg, L-Gln	5, 5	42
[62]	Poland	16.94	Low-CP	Corn-wheat-soybean	Ross 308	Male	≥21	Glycine	4.2	35
[63]	Malaysia	20.26	Standard	Corn-soybean	Cobb 500	-	≥21	L-Arginine	2.5	42
[64]	South Korea	19.25	Low-CP	Corn-soybean	Ross 308	Mixed sex	≥21	Glycine	4, 8, 10.6	17
[65]	Iran	23.36	Standard	Corn-soybean	Ross 308	-	≤21	L-Glutamine	10, 20	14
[66]	Iran	19.82	Low-CP	Corn-soybean	Ross 308	Male	≥21	L-Arginine	2,4	37
[67]	Iran	18.2	Low-CP	Corn-soybean	Ross 308	Male	≥21	L-Arginine	21	28
[67]	India	17.61	Low-CP	-	Cobb-430Y	-	≥21	Glycine	6.4, 8.1, 6.3	42
[68]	China	22.2	Standard	Corn-soybean	Arbor Acres	Mixed sex	≤ 21	L-Glutamine	5, 10	21
[36]	China	20.66	Standard	Corn-corn gluten soybean meal	Arbor Acres	Mixed sex	≥21	L-Arginine	4.5, 9, 13.5, 18	42
[69]	China	21.94	Standard	Corn-corn gluten soybean meal	Arbor Acres	Male	≤21	L-Arginine	3	21

Abbreviations: Low- CP-low crude protein; Arg-arginine; Gln-glutamine; Gly-glycine. ≤21 = growth phase less than or equal to 21 days; ≥21 = growth phase greater than or equal to 21 days. ^a^ = Calculated as the basal diet’s chemical composition crude protein adjusted for each feeding phase. ^b^ = Studies showing two or three of the evaluated FAA indicate independent supplementation of the basal diet with either arginine, glutamine, or glycine as intervention.

**Table 2:** Publication Bias and Trim-Fill Analysis of Growth, Jejunal morphology, and immune organ response variables.

Variable	Begg’s	MS	Adjusted SMD [95% CIs]	*p*-Value	Prediction Interval
ADFI	0.3453	*	*	*	*
BWG	<0.0001	14	0.14 [−0.54; 0.82]	0.6871	−5.00; 5.28
FCR	<0.0001	9	−0.23 [−0.44 −0.02]	0.0296	−1.66; 1.20
VH	0.0002	5	0.27 [−0.60; 1.15]	0.5445	−3.36; 3.91
CD	0.0020	2	−0.77 [−1.38; −0.17]	0.0117	−2.77; 1.22
VH-to-CD	0.0013	3	0.84 [0.33; 1.34]	0.0011	−0.68; 2.35
Spleen	0.5403	*	*	*	*
Bursa	0.2758	*	*	*	*

Abbreviation: MS = missing studies imputed; ADFI = average daily feed intake; BWG = body weight gain; FCR = feed conversion ratio; VH = villus height; CD = crypt depth; VH-to-CD = villus height to crypt depth ratio. * = Trim and Fill analysis not applicable. The ileal morphology variable did not meet the minimum number of studies required for a publication bias analysis; therefore, a trim-and-fill analysis was not performed. Funnel plot visualizations of outcome variables with publication bias are presented in Supplementary Figure S10.

**Table 3:** Functional Amino Acid effects on broiler immune organ development and intestinal histomorphometry response.

	Random Effects Model				Heterogeneity	
Variable	N	SMD	95% CI	*p*-Value	Q	*I* ^2^
Jejunal Morphology						
VH	12	1.06	0.36; 1.76	0.0030	32.10	65.70
CD	12	−0.97	−1.46; −0.47	0.0001	20.70	46.90
VH-to-CD	12	1.05	0.64; 1.45	<0.0001	16.42	33.00
Ileal Morphology						
VH	8	1.10	0.51; 1.69	0.0002	8.95	21.80
CD	8	−0.54	−1.01; −0.08	0.0222	5.53	0.00
VH-to-CD	8	1.07	0.55; 1.58	<0.0001	7.04	0.50
Immune Organ						
Spleen	13	0.24	−0.16; 0.64	0.2483	20.02	40.10
Bursa	11	0.31	−0.12; 0.73	0.1575	13.48	25.80

Abbreviation: ADFI = average daily feed intake; BWG = body weight gain; FCR = feed conversion ratio; VH = villus height; CD = crypt depth; VH-to-CD = villus height to crypt depth ratio; N = number of studies; SMD = standardized mean difference; *Q* = test statistic for heterogeneity; *I*^2^ = proportion of the total variation in effect size across studies. Forest plots showing standardized mean difference effects are presented in Supplementary Figures S1–S9.

**Table 4:** Subgroup-Specific Differences in Average Daily Feed Intake.

					Heterogeneity		
Moderator	Subgroup	N	SMD [95% CI]	*p*-Value	*I*^2^ (%)	*p*-Value	R^2^ (%)
Amino acid type	L-Arginine	21	−0.26 [−0.69; 0.17]	0.5361	93.20	<0.0001	0.00
	L-Glutamine	4	−0.15 [−0.78; 0.48]		92.20	<0.0001	
	Glycine	15	−0.02 [−0.13; 0.09]		55.90	0.0044	
CP formulation	Standard	16	−0.30 [−0.85; 0.26]	0.4321	94.10	<0.0001	1.30
	Low-CP	24	−0.08 [−0.14; −0.03]		78.30	<0.0001	
Growth phase, d	≤21	9	0.14 [0.04; 0.24]	0.0162	0.00	0.8211	4.60
	≥21	31	−0.23 [−0.52; 0.06]		91.30	<0.0001	
Strain	Arbor Acres	14	−0.24 [−0.82; 0.34]	<0.0001	92.90	<0.0001	17.10
	Cobb 500	7	0.10 [−0.10; 0.29]		60.00	0.0202	
	Ross 308	13	−0.19 [−0.37; −0.02]		75.60	<0.0001	
	Cobb-430Y	3	−0.03 [−0.30; 0.23]		38.60	0.1961	
	Ross × Ross	2	0.47 [−0.45; 1.39]		—	—	
	Ross	1	−1.93 [−2.41; −1.46]		—	—	

Abbreviation: N = number of studies; SMD = standardized mean difference; I^2^ = proportion of the total variation in effect size across studies; R^2^ = amount of heterogeneity accounted for by the moderator.

**Table 5:** Comparison of Body Weight Gain by Subgroup Characteristics.

					Heterogeneity		
Moderator	Subgroup	N	SMD [95% CI]	*p*-Value	*I*^2^ (%)	*p*-Value	R^2^ (%)
Amino acid type	L-Arginine	21	0.62 [0.54; 0.70]	0.0185	95.10	<0.0001	10.60
	L-Glutamine	4	3.25 [−0.84; 7.35]		99.50	<0.0001	
	Glycine	15	0.24 [0.08; 0.39]		76.70	<0.0001	
CP formulation	Standard	16	2.18 [0.89; 3.47]	0.0037	98.30	<0.0001	15.90
	Low-CP	24	0.26 [0.08; 0.43]		84.40	<0.0001	
Growth phase, d	≤21	9	1.86 [0.02; 3.69]	0.2581	98.60	<0.0001	4.90
	≥21	31	0.75 [0.26; 1.26]		94.70	<0.0001	
Strain	Arbor Acres	14	1.90 [0.72; 3.07]	<0.0001	98.10	<0.0001	14.20
	Cobb 500	7	0.25 [−0.01; 0.50]		76.10	0.0003	
	Ross 308	13	0.12 [−0.15; 0.39]		89.80	<0.0001	
	Cobb-430Y	3	0.69 [0.47; 0.91]		7.20	0.3405	
	Ross × Ross	2	0.62 [0.25; 0.99]		62.20	0.1036	
	Ross	1	8.17 [6.96; 9.40]		—	—	

Abbreviation: N = number of studies; SMD = standardized mean difference; I^2^ = proportion of the total variation in effect size across studies; R^2^ = amount of heterogeneity accounted for by the moderator.

**Table 6:** Effect of Treatment on Feed Conversion Ratio in Defined Subgroups.

					Heterogeneity		
Moderator	Subgroup	N	SMD [95% CI]	*p*-Value	*I*^2^ (%)	*p*-Value	R^2^ (%)
Amino acid type	L-Arginine	20	−0.64 [−0.94; −0.34]	0.0002	90.50	<0.0001	31.20
	L-Glutamine	4	−0.03 [−0.18; 0.12]		0.00	0.7254	
	Glycine	15	−0.33 [−0.46; −0.22]		61.40	<0.0001	
CP formulation	Standard	16	−0.48 [−0.79; −0.18]	0.7935	86.20	<0.0001	0.50
	Low-CP	23	−0.44 [−0.63; −0.24]		85.00	<0.0001	
Growth phase, d	≤21	9	−0.20 [−0.35; −0.05]	0.0113	87.60	<0.0001	2.80
	≥21	30	−0.53 [−0.74; −0.33]		48.90	0.0477	
Strain	Arbor Acres	14	−0.49 [−0.82; −0.17]	<0.0001	84.70	<0.0001	12.20
	Cobb 500	7	−0.19 [−0.39; 0.01]		62.20	0.0145	
	Ross 308	12	−0.52 [−0.86; −0.19]		90.20	<0.0001	
	Cobb-430Y	3	−0.77 [−0.99; −0.56]		0.00	0.7124	
	Ross × Ross	2	−0.01 [−0.29; 0.27]		37.80	0.2049	
	Ross	1	−0.99 [−1.41; −0.58]		—	—	

Abbreviation: N = number of studies; SMD = standardized mean difference; I^2^ = proportion of the total variation in effect size across studies; R^2^ = amount of heterogeneity accounted for by the moderator.

**Table 7:** Comparison of Jejunal Villus Height in Broilers According to Subgroup Characteristics.

					Heterogeneity		
Moderator	Subgroup	N	SMD [95% CI]	*p*-Value	*I*^2^ (%)	*p*-Value	R^2^ (%)
Amino acid type	L-Arginine	2	0.28 [−1.46; 2.02]	0.0130	75.20	0.0447	27.10
	L-Glutamine	7	1.85 [0.94; 2.76]		50.10	0.0613	
	Glycine	3	0.19 [−0.46; 0.84]		21.10	0.2816	
CP formulation	Standard	7	1.72 [0.55; 2.89]	0.0423	75.20	0.0005	12.80
	Low-CP	5	0.39 [−0.14; 0.92]		10.90	0.3440	
Growth phase, d	≤21	5	1.72 [0.55; 2.89]	0.0423	10.90	0.3440	12.80
	≥21	7	0.39 [−0.14; 0.92]		75.20	0.0005	
Strain	Arbor Acres	1	−0.53 [−1.54; 0.47]	0.0175	—	<0.0001	25.20
	Ross 308	9	1.36 [0.48; 2.25]		68.10	<0.0001	
	Ross × Ross	2	0.89 [−0.06; 1.85]		0.00	0.2049	

Abbreviation: N = number of studies; SMD = standardized mean difference; I^2^ = proportion of the total variation in effect size across studies; R^2^ = amount of heterogeneity accounted for by the moderator.

**Table 8:** Meta-Regression of Growth Performance Parameters (ADFI, BWG, FCR) and Jejunal Villus Height.

	Test of Moderators		Mixed-Effects Model Estimate		
Variable	QM [df]	*p*-Value	^1^ Intercept [SE]	Moderator [SE]	R^2^ (%)
Amino Acid Dose					
ADFI	3.40 [1]	0.0652	−0.14 [0.11]	0.04 [0.02]	6.00
BWG	0.07 [1]	0.7921	1.12 [0.51]	−0.02 [0.06]	0.00
FCR	0.58 [1]	0.4451	−0.45 [0.08]	0.02 [0.02]	0.00
Jejunal VH	3.81 [1]	0.0510	1.06 [0.31]	0.10 [0.05]	37.50
Supplementation Duration, days					
ADFI	0.06 [1]	0.8081	−0.14 [0.12]	−0.003 [0.01]	0.00
BWG	0.05 [1]	0.8243	1.02 [0.30]	0.01 [0.03]	0.00
FCR	4.23 [1]	0.0396	−0.46 [0.08]	−0.02 [0.01]	9.00
Jejunal VH	0.53 [1]	0.4663	1.10 [0.38]	−0.03 [0.04]	0.00
Calculated CP%, DM					
ADFI	1.08 [1]	0.2992	−0.14 [0.11]	−0.06 [0.06]	0.00
BWG	9.63 [1]	0.0019	1.01 [0.27]	0.41 [0.13]	18.70
FCR	0.53 [1]	0.4684	−0.45 [0.08]	0.03 [0.04]	0.00
Jejunal VH	3.67 [1]	0.0553	1.13 [0.34]	0.28 [0.15]	20.00

QM = test of moderators; R^2^ = amount of heterogeneity accounted for by the moderator. ^1^ = Centered intercept.

## Data Availability

The raw datasets for the contributions presented in the study are shown in the forest plots included in the article and Supplementary Materials; further inquiries can be directed to the corresponding author.

## Supplementary Materials

The following supporting information can be downloaded at https://www.mdpi.com/article/10.3390/ani16081207/s1: Figure S1: Weighted bar plots of the distribution of risk-of-bias judgements within each bias domain; Figures S2–S4: Jejunal Morphology; Figures S5–S7: Ileal Morphology; Figures S8 and S9: Immune organ weight; Figure S10: Funnel plots: BWG (A), FCR (B), jejunal VH (C), jejunal CD (D), and jejunal VH-to-CD ratio (D).

## Abbreviations

The following abbreviations are used in this manuscript:

FAA	Functional amino acids
SYRCLE	Systematic review center for laboratory animal experimentation
PRISMA	Preferred reporting items for systematic reviews and meta-analyses
PICO	Population intervention comparator outcome
CP	Crude protein
Arg	L-arginine
Gln	L-glutamine
Gly	Glycine
ADFI	Average daily feed intake
BWG	Body weight gain
FCR	Feed conversion ratio
SMD	Standardized mean difference
CI	Confidence interval
QM	Test of moderators
VH	Villus height
CD	Crypt depth
VH-to-CD	Villus height to crypt ratio
