# Supplementary material for: Functional Amino Acid Supplementation Drives Early Growth and Gut Maturation in Broilers: A Meta-Analysis
Source: Animals (Basel). 2026 Apr 15;16(8):1207. doi: 10.3390/ani16081207 (PMC13114196; doi:10.3390/ani16081207)
Supplement: Supplementary file 1 [file animals-16-01207-s001.zip › animals-4215996-supplementary.pdf]

Functional Amino Acid Supplementation Drives Early Growth and Gut Maturation in Broilers: A Meta-Analysis

Emmanuel Nuamah, Utibe Mfon Okon, Jongryun Kim, Guybong Song, Darae Kang, Hak-kyo Lee, Kwanseob Shim

Supplementary Figures

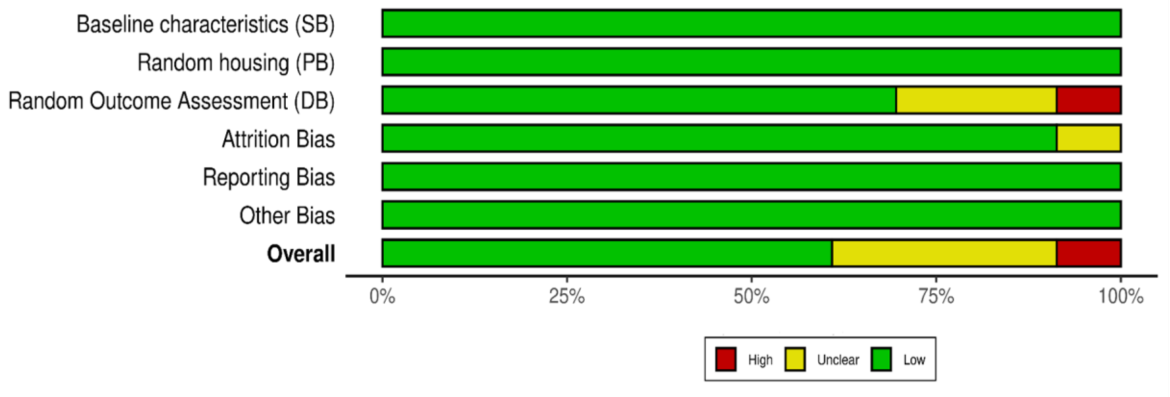

Supplementary Figure S1. Weighted bar plots of the distribution of risk-of-bias judgements within each bias domain.

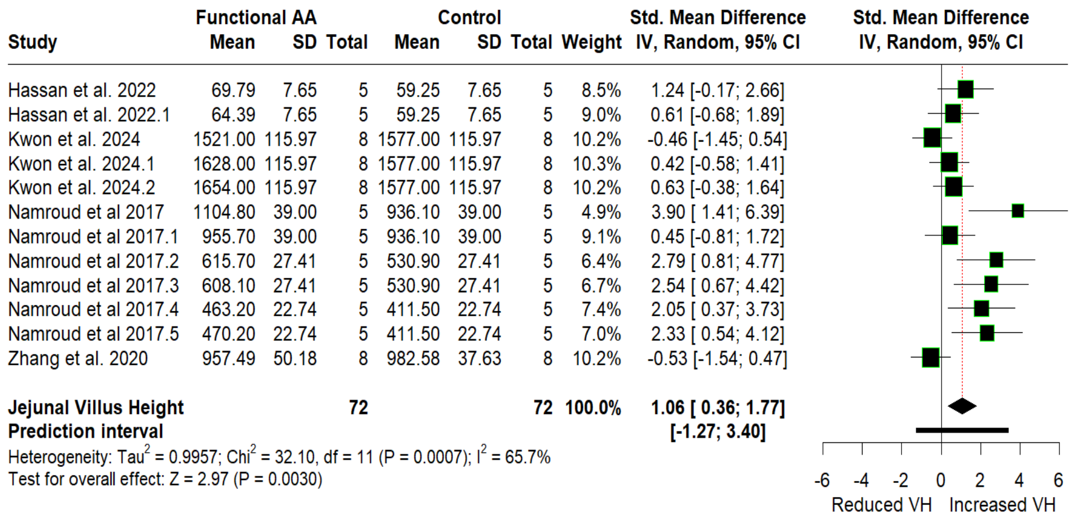

Supplementary Figure S2. The forest plot shows the SMD and 95% CI for post-hatch supplementation with L-Arginine, L-Glutamine, and Glycine on broiler jejunal VH.

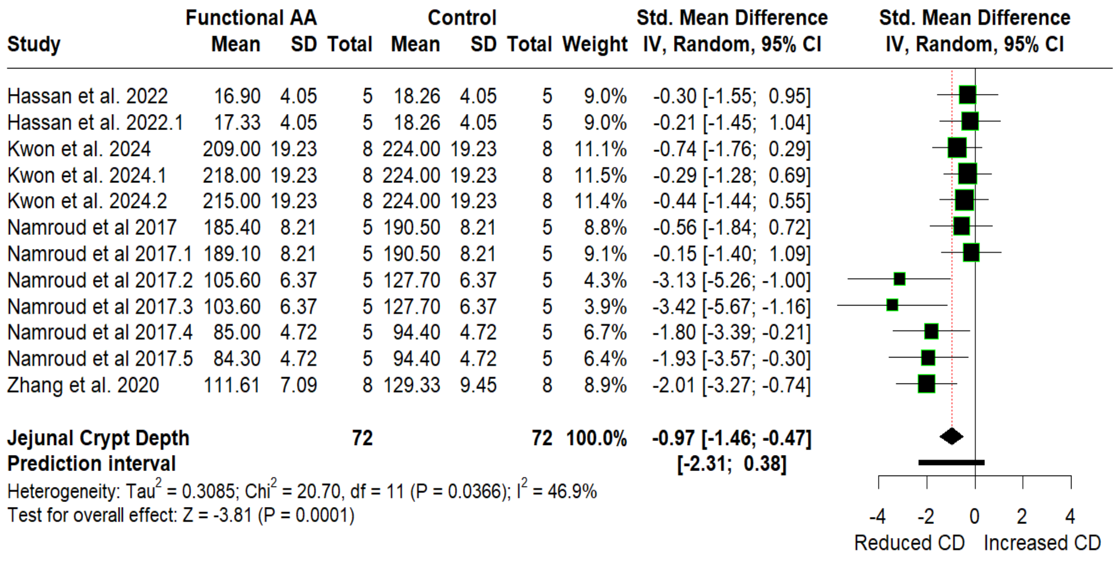

Supplementary Figure S3. The forest plot shows the SMD and 95% CI for post-hatch L-Arginine, L-Glutamine, and Glycine supplementation effects on jejunal CD in broilers.

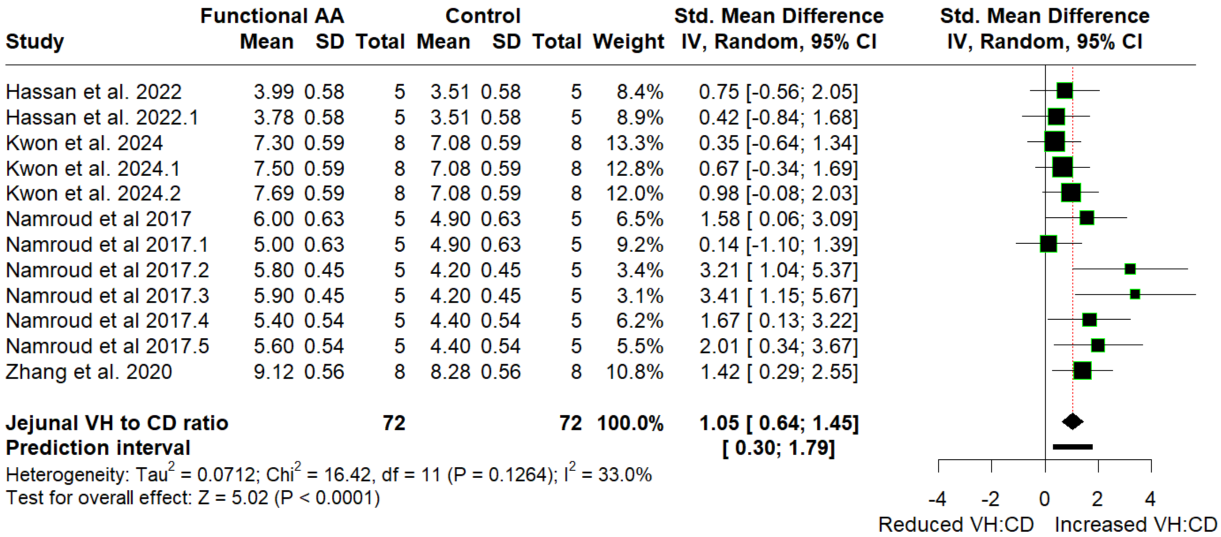

Supplementary Figure S4. The forest plot illustrates the SMD and corresponding 95% CI for the effects of post-hatch supplementation with L-Arginine, L-Glutamine, and Glycine on the jejunal VH-to-CD ratio in broilers.

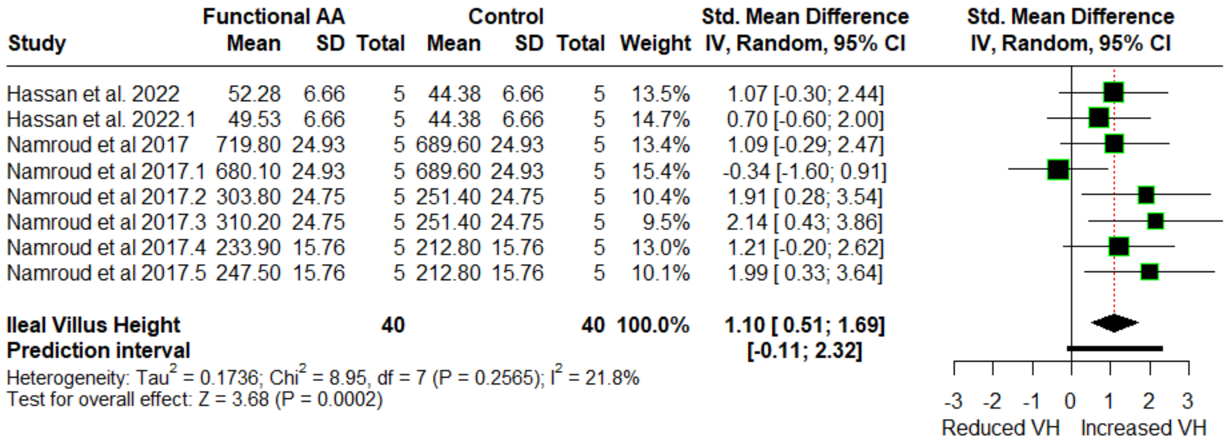

Supplementary Figure S5. The forest plot illustrates the SMD and corresponding 95% CI for the effects of post-hatch L-Arginine, L-Glutamine, and Glycine supplementation on ileal VH in broilers.

Study	Functional AA Mean	Functional AA SD	Functional AA Total	Control Mean	Control SD	Control Total	Weight	Std. Mean Difference IV, Random, 95% CI
Hassan et al. 2022	13.45	3.78	5	15.01	3.78	5	13.6%	-0.37 [-1.63; 0.88]
Hassan et al. 2022.1	14.69	3.78	5	15.01	3.78	5	14.0%	-0.08 [-1.32; 1.16]
Namroud et al 2017	95.20	10.29	5	96.70	10.29	5	14.0%	-0.13 [-1.37; 1.11]
Namroud et al 2017.1	93.70	10.29	5	96.70	10.29	5	13.8%	-0.26 [-1.51; 0.98]
Namroud et al 2017.2	75.10	3.32	5	80.20	3.32	5	10.1%	-1.39 [-2.84; 0.07]
Namroud et al 2017.3	73.60	3.32	5	80.20	3.32	5	8.5%	-1.79 [-3.38; -0.21]
Namroud et al 2017.4	76.50	3.18	5	77.90	10.29	5	13.9%	-0.17 [-1.41; 1.08]
Namroud et al 2017.5	70.30	3.18	5	77.90	10.29	5	12.1%	-0.90 [-2.24; 0.44]
**Ileal Crypt Depth**			**40**			**40**	**100.0%**	**-0.54 [-1.01; -0.08]**
**Prediction interval**								**[-1.10; 0.02]**

Heterogeneity: $Tau^2 = 0$; $Chi^2 = 5.53$, df = 7 (P = 0.5957); $I^2 = 0.0\%$
Test for overall effect: Z = -2.29 (P = 0.0222)

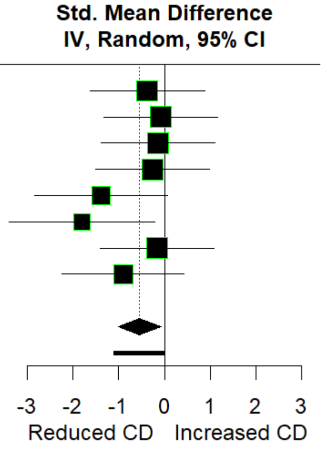

Supplementary Figure S6. The forest plot illustrates the SMD and corresponding 95% CI for the effects of post-hatch supplementation with L-Arginine, L-Glutamine, and Glycine on ileal CD in broilers.

Study	Functional AA Mean	Functional AA SD	Functional AA Total	Control Mean	Control SD	Control Total	Weight	Std. Mean Difference IV, Random, 95% CI
Hassan et al. 2022	3.49	0.40	5	3.09	0.40	5	14.1%	0.90 [-0.43; 2.24]
Hassan et al. 2022.1	3.85	0.40	5	3.09	0.40	5	10.4%	1.72 [0.15; 3.28]
Namroud et al 2017	7.60	0.85	5	7.20	0.85	5	15.8%	0.42 [-0.84; 1.69]
Namroud et al 2017.1	7.30	0.85	5	7.20	0.85	5	16.3%	0.11 [-1.13; 1.35]
Namroud et al 2017.2	4.00	0.47	5	3.10	0.47	5	10.4%	1.73 [0.16; 3.29]
Namroud et al 2017.3	4.20	0.47	5	3.10	0.47	5	8.8%	2.11 [0.41; 3.82]
Namroud et al 2017.4	3.10	0.40	5	2.70	0.40	5	14.1%	0.90 [-0.43; 2.24]
Namroud et al 2017.5	3.50	0.40	5	2.70	0.40	5	10.1%	1.81 [0.21; 3.40]
**Ileal VH:CD Ratio**			**40**			**40**	**100.0%**	**1.07 [0.55; 1.58]**
**Prediction interval**								**[0.38; 1.75]**

Heterogeneity: $Tau^2 = 0.0165$; $Chi^2 = 7.04$, df = 7 (P = 0.4251); $I^2 = 0.5\%$
Test for overall effect: Z = 4.09 (P < 0.0001)

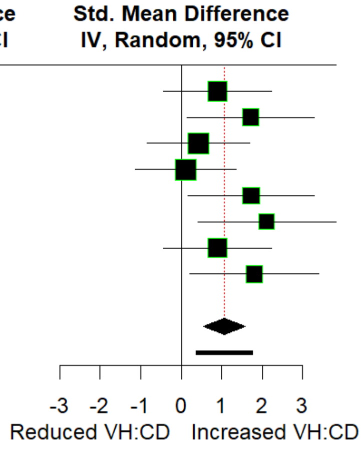

Supplementary Figure S7. The forest plot illustrates the SMD and corresponding 95% CI for the effects of post-hatch L-Arginine, L-Glutamine, and Glycine supplementation on ileal VH:CD ratio in broilers.

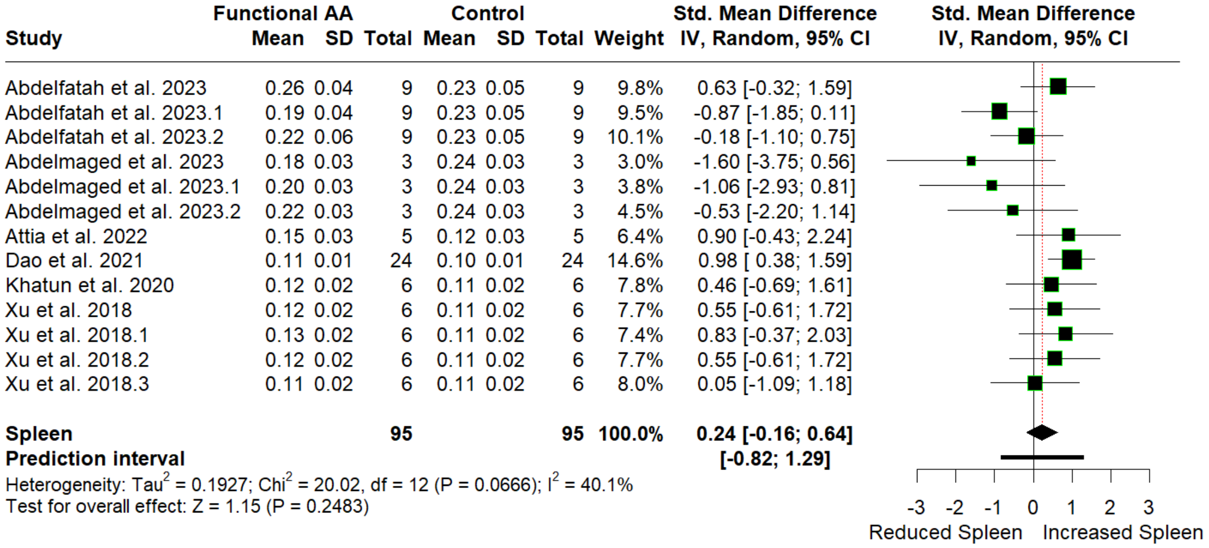

Supplementary Figure S8. The forest plot illustrates the SMD and corresponding 95% CI for the effects of post-hatch supplementation with L-Arginine, L-Glutamine, and Glycine on relative spleen weight in broilers.

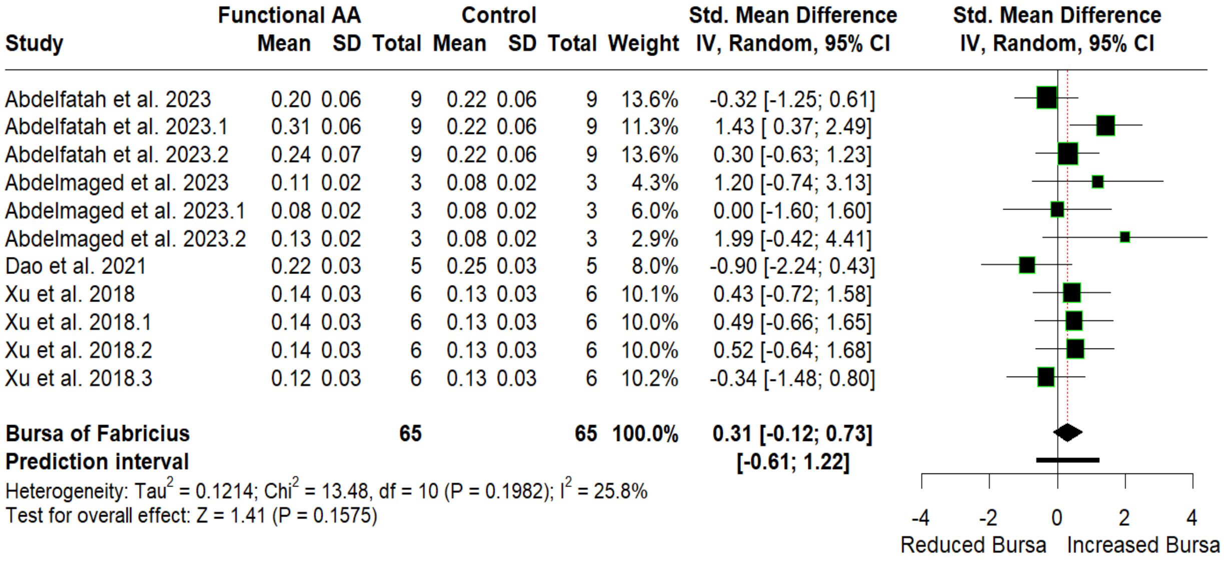

Supplementary Figure S9. The forest plot illustrates the SMD and corresponding 95% CI for the effects of post-hatch L-Arginine, L-Glutamine, and Glycine supplementation on relative bursa of Fabricius weight in broilers.

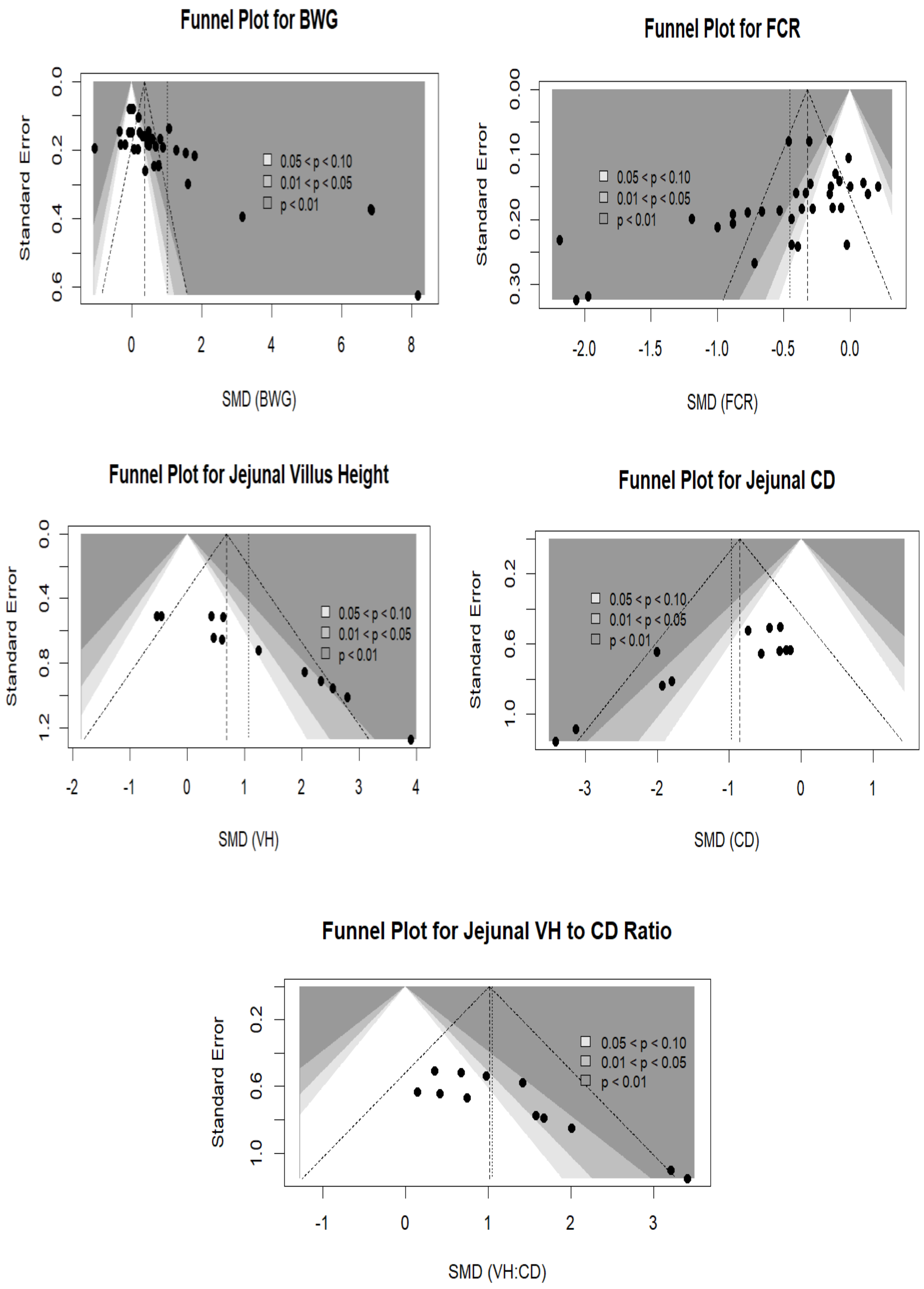

Supplementary Figure S10. Funnel plots: BWG (A), FCR (B), jejunal VH (C), jejunal CD (D), and jejunal VH-to-CD ratio (D).
